# Supplementary material for: Core Outcome Measurement Instruments for Clinical Trials of Total Knee Arthroplasty: A Systematic Review
Source: J Clin Med. 2020 Jul 30;9(8):2439. doi: 10.3390/jcm9082439 (PMC7463550; doi:10.3390/jcm9082439)
Supplement: Supplementary file 1 [file jcm-09-02439-s001.pdf]

Journal of
Clinical Medicine

MDPI

Review

Core Outcome Measurement Instruments for Clinical Trials of Total Knee Arthroplasty: A Systematic Review

Vivien Reynaud [1,*], Anargyros Verdilos [2], Bruno Pereira [3], Stéphane Boisgard [4], Frédéric Costes [1] and Emmanuel Coudeyre [5]

1 Plateforme d'Exploration de la Mobilité, INRAE, UNH, CHU Clermont-Ferrand, Université Clermont Auvergne, F-63000 Clermont–Ferrand, France; fcostes@chu-clermontferrand.fr
2 Plateforme d'Exploration de la Mobilité, CHU Clermont-Ferrand, Université Clermont Auvergne, F-63000 Clermont–Ferrand, France; averdilos@chu-clermontferrand.fr
3 Unité de Biostatistique, CHU Clermont-Ferrand, Direction Recherche Clinique et Innovation, Université Clermont Auvergne, F-63000 Clermont–Ferrand, France; bpereira@chu-clermontferrand.fr
4 Service d'Orthopédie Traumatologie, CNRS, SIGMA Clermont, CHU Clermont-Ferrand, Université Clermont Auvergne, ICCF, F-63000 Clermont–Ferrand, France; sboisgard@chu-clermontferrand.fr
5 Service de Médecine Physique et de Réadaptation, INRAE, UNH, CHU Clermont-Ferrand, Université Clermont Auvergne, F-63000 Clermont–Ferrand, France; ecoudeyre@chu-clermontferrand.fr
* Correspondence: vreynaud@chu-clermontferrand.fr; Tel.: +33-47375-0900

Received: 29 June 2020; Accepted: 27 July 2020; Published: date

Abstract: (1) Background: We have updated knowledge of the psychometric qualities of patient-reported outcome measures and, for the first time, systematically reviewed and compared the psychometric qualities of physical tests for patients with knee osteoarthritis who are undergoing total knee arthroplasty. This work was conducted to facilitate the choice of the most appropriate instruments to use in studies and clinical practice. (2) Methods: A search of medical databases up to December 2019 identified the studies and thus the instruments used. The quality of the measurement properties was assessed by the Bot et al. criteria. (3) Results: We identified 20 studies involving 25 instruments. Half of the instruments were questionnaires (n = 13). Among the condition-specific instruments, the Oxford knee score, Knee injury and Osteoarthritis Outcomes Score, and the Western Ontario and McMaster Universities Osteoarthritis index had the highest overall scores. Concerning generic tools, the Medical Outcomes Study Short-Form 36 (SF-36) or SF-12 obtained the highest overall score. For patient-specific tools, the Hospital Anxiety and Depression Scale ranked the highest. Some physical tests seemed robust in psychometric properties: 6-min Walk Test, five times Sit-To-Stand test, Timed Up and Go test strength testing of knee flexor/extensor by isometric or isokinetic dynamometer and Pressure Pain Threshold. (4) Conclusion: To make stronger recommendations, key areas such as reproducibility, responsiveness to clinical change, and minimal important change still need more rigorous evaluations. Some promising physical tests (e.g., actimetry) lack validation and require rigorous studies to be used as a core set of outcomes in future studies.

Keywords: systematic review; total knee arthroplasty; primary total knee arthroplasty; clinical outcomes; functional outcomes; patient-reported outcome measures

1. Introduction

Knee osteoarthritis (OA) is a common degenerative osteoarticular disease, associating pain, stiffness, and loss of mobility. It affects 2% to 10% of men and 1.6% to 15% of women over age 40

depending on geographical areas and definitions of pathology [1]. According to a US study, 45% of adults over age 45 will have knee OA before the age of 85 [2]. OA has important consequences for quality of life, disability and mobility [1] and is associated with an increase in mortality [3]. It is a heterogeneous disorder characterized by various etiological factors, pathophysiological pathways, clinical phenotypes and prognosis [4]. Indeed, more than half of patients with radiographic OA are not symptomatic; for 50% to 70%, disease will not worsen radiographically in 2 years; for 27%, disease will progress slowly or moderately; and for 2%, disease will progress rapidly, known as rapidly destructive OA of the knee [5].

We lack long-term effective pharmacological treatment for OA, especially for frail patients, but nonpharmacological treatments (e.g., physical therapy, surgery) have been found effective [6–8]. The use of surgery is frequent: the 2010 prevalence of knee replacement in the total US population was 1.52%, with 4.7 million individuals undergoing total knee replacement [9]. In France in 2011, 86,000 knee replacements were performed, increasing by more than 33.0% between 2008 and 2013 [10].

In end-stage knee OA, total knee arthroplasty (TKA) is an effective intervention to reduce pain and improve function for most patients. However, after TKA, some patients still experience pain, loss of function, deficient muscle strength or reduced walking speed.

Outcomes of patients with knee OA undergoing or after TKA are evaluated with many different instruments used both in clinical practice and in research. The Outcome Measures in Rheumatology Clinical Trials (OMERACT) group defined a core set of outcome dimensions for clinical studies in knee OA at its medical stage: pain, physical function (the performance of daily activities), and patient global assessment [11,12]. In the same way, the International Classification of Functioning, Disability and Health (ICF) defined a core set of outcome dimensions: impairments of body functions and structures, activity limitations and participation restriction, and environmental factors [13]. These comply with the recommendations of several guidelines for outcome measurement in OA trials (European League Against Rheumatism [14], Osteoarthritis Research Society International [15], US Food and Drug Administration [11], and Slow-acting Drugs in Osteoarthritis [16]). However, these guidelines differ in their recommendations for the use of specific instruments [14,15] or simply lack any recommendations in this regard for knee OA undergoing or after TKA.

Many instruments, such as patient-reported outcome measures (PROMs) and physical tests, are available to assess the outcome dimensions of the OMERACT or ICF. However, which instruments are the most appropriate is unclear [17]. In fields like satisfaction after TKA, we lack an objective assessment tool to evaluate the impact of TKA and to better understand the heterogeneity between a patient's post-surgery status [18]. Instrument selection should depend on the instrument's psychometric qualities and on practical considerations (e.g., time to complete, ease of scoring or use, mode of administration and distribution, meaning its validity by use, whatever its metrological properties). Psychometric qualities refer mainly to the validity and reliability of the measuring tool [19]. Validity meaning *"the extent to which an instrument measures what it is intended to measure"* and reliability the fact that the instrument is free of error [19].

Several reviews of OA tools at the medical stage have been published, and a special issue on outcome measurements was published by *Arthritis Care & Research* [11, 21–23]. Two separate studies [20,21] concluded that the Western Ontario and McMaster Universities Osteoarthritis Index (WOMAC), the Oxford Knee Score (OKS) and the Medical Outcomes Study Short-Form 36 (SF-36) could be recommended as primary measures in treatment studies knowing that certain key areas such as reproducibility, responsiveness to clinical change, and minimal important change needed more rigorous evaluation to make stronger recommendations. However, none of these reviews focused on physical tests. In addition, an update 10 years later seems necessary in order to allow a complete overview of available instruments for patients with knee OA undergoing or after TKA. To assess the selected instruments, data on the descriptive and psychometric qualities of each instrument will be collected and rated by using the same checklist [22,23].

The objective of this study was to give an overview of a core set of PROMs as well as physical tests used for clinical trials of individuals with knee OA undergoing TKA. This review will facilitate

the choice of the most appropriate measurement instruments (PROMs and physical tests) for studies and clinical practice of patients with knee OA who are undergoing TKA.

2. Experimental Section

The review and analysis were conducted and reported in accordance with the Preferred Reporting Items for Systematic Reviews and Meta-analyses (PRISMA) [24]. The systematic review protocol was registered in PROSPERO (CRD42020161878). Information regarding study selection, search strategy, inclusion and exclusion criteria, risk of bias and quality of evidence, data extraction, and psychometric qualities are shown in detail in the Supplementary Materials.

2.1. Study Selection

2.1.1. Search Strategy

PubMed, EMBASE, Web of Science, Cochrane Central Register of Controlled Trials and CINAHL databases were systematically searched for articles published from 2014 through December 2019. The broad computerized search strategy was built on key words for patients with OA of the knee undergoing TKA; search strategy for outcome assessment; and search strategy for control trial. Search terms are listed in Research Algorithm S1. Selection of articles was based on their title and abstract and was decided by two independent reviewers (VR and AV). Their inclusion was then decided after reading the full article by the same two independent reviewers. The full article was read by two other independent reviewers (FC and EC) in case of doubt or concern and if necessary, a third reviewer (SB) resolved disagreements.

Regarding clinimetric studies, a search following the same process and hierarchy was made using PubMed database. In addition, references of the retrieved articles were screened for relevant articles.

2.1.2. Inclusion and Exclusion Criteria

Inclusion criteria were as follows: (1) Study design: all randomized controlled trials (RCTs) or clinical controlled trials (CCTs); (2) Patients: individuals with OA of the knee undergoing TKA; (3) Intervention: articles focused on unilateral and primary TKA (not total hip arthroplasty) because those patients were considered different patient populations; (4) Outcomes: data measured before and after TKA. Given the large number of articles published on this topic and considering that a certain number of outcomes have been used only recently (last 5 years), we included only articles published since 2014 (≤5 years); (5) Instruments: all outcomes (PROMs and physical tests) used as primary or secondary criteria. Finally, inclusion criteria at the level of clinimetric studies were as follows: article's main focus was the psychometric evaluation of the instrument (since no checklist to rate psychometric evaluations based on item response theory [IRT] is currently available, only psychometric evaluations involving usual test theory were included and evaluations based on IRT were excluded); data for patients with knee OA undergoing TKA were published independently in case of mixed populations (e.g., patients with OA undergoing total hip arthroplasty and patients with OA undergoing TKA); and results had been published in English as a full report.

2.2. Quality Assessment and Data Extraction

The Physiotherapy Evidence Database (PEDro) scale [25] was used to assess risk of bias within studies. At the level of clinimetric studies, to facilitate comparisons with other studies from 10 years ago [7,26], fundamentally the same checklist of specific criteria for quality assessment of instruments was used (Table S1 for self-assessment tools and Table S2 for physical tests). This checklist was initially developed by Bot et al. [27] based on the work by Lohr et al. [28], the Scientific Advisory Committee of the Medical Outcomes Trust guidelines and the checklist developed by Bombardier and Tugwell [29]. All qualities were rated as positive, doubtful, or negative. If no or insufficient information was available, no rating was given. Two reviewers (VR and AV) independently assessed

the psychometric qualities of each instrument. Disagreements between reviewers were resolved by discussion. The highest rating was assigned when ≥2 studies were found on the same psychometric qualities of the same instrument involving the same population.

2.3. Characteristics of the Instruments

The descriptive data recovered provide information about the target population, domain assessed, format of the instruments and mandatory equipment for physical tests (Table A1).

2.4. Psychometric Qualities

Psychometric qualities (validity, reproducibility, responsiveness, and interpretability) were assessed for each instrument in this specific population, namely knee OA, and more precisely TKA according to the COnsensus-based Standards for the selection of health status Measurement Instruments (COSMIN) recommendations [30]. See supplementary material for more detailed information (Table S1 for self-assessment tools and Table S2 for physical tests).

2.5. Overall Quality

As Bot et al. described [31,27], overall score of the instruments was obtained by adding the number of positive ratings for each psychometric quality (Table S1 for self-assessment tools and Table S2 for physical tests).

2.6. Statistical Analysis

Except the calculation of the agreement kappa coefficient between two reviewers, statistical analyzes were exclusively descriptive and involved use of Microsoft Office Excel 2019 (Microsoft Corp., Redmond, WA, USA). To assess the concordance between the two reviewers proofreading, we calculate a kappa coefficient using Stata v13 (StataCorp, College Station, TX, USA), considering a categorical criterion, and the number of modalities of the variable studied.

3. Results

3.1. Study Selection

A flow chart detailing the study selection process is shown in Figure 1. The initial searches returned a total of 2339 articles; 313 were duplicates. Titles and abstracts of the retrieved articles were assessed for suitability, leading to the retrieval of 105 full texts. Of these, 85 did not fulfill the inclusion criteria and reports for the remaining 20 studies were analyzed. The kappa statistic between two reviewers (VR and AV) was 0.74, which indicated good agreement [32].

3.2. Study Characteristics

The included studies involved 1997 participants (1005 interventions and 992 controls). For 12 (60%) studies, the design was randomized controlled trial (RCTs). A summary of the included trials is shown in Table 1.

3.3. Description of Outcomes

All included outcomes (n = 25) are presented in Table 2. Half were questionnaires (n = 13, 52%) developed to assess pain (n = 8, 61%) and/or physical function (n = 6, 46%) in separate (sub)scales. These questionnaires could be divided into 3 categories: those assessing a specific condition (knee OA; n = 5, 38%), a patient specificity such as anxiety or depression (n = 4, 31%) and generic outcomes (n = 4, 31%) (Table 3). Other outcomes were physical tests (n = 12, 48%) mostly developed to assess functional mobility (n = 6, 50%). We could distinguish analytical tests (n = 5, 42%) and performance-based tests (n = 7, 58%) such as walking tests (n = 4, 33%), sit-to-stand tests (n = 2, 17%) and stair climbing tests (n = 1, 8%) (Table 3). Among the 25 outcomes, 13 (52%) were used as a primary outcome

in RCTs and 21 (84%) as a secondary outcome (including 9 [36%] both primary and secondary outcomes) (Table 2).

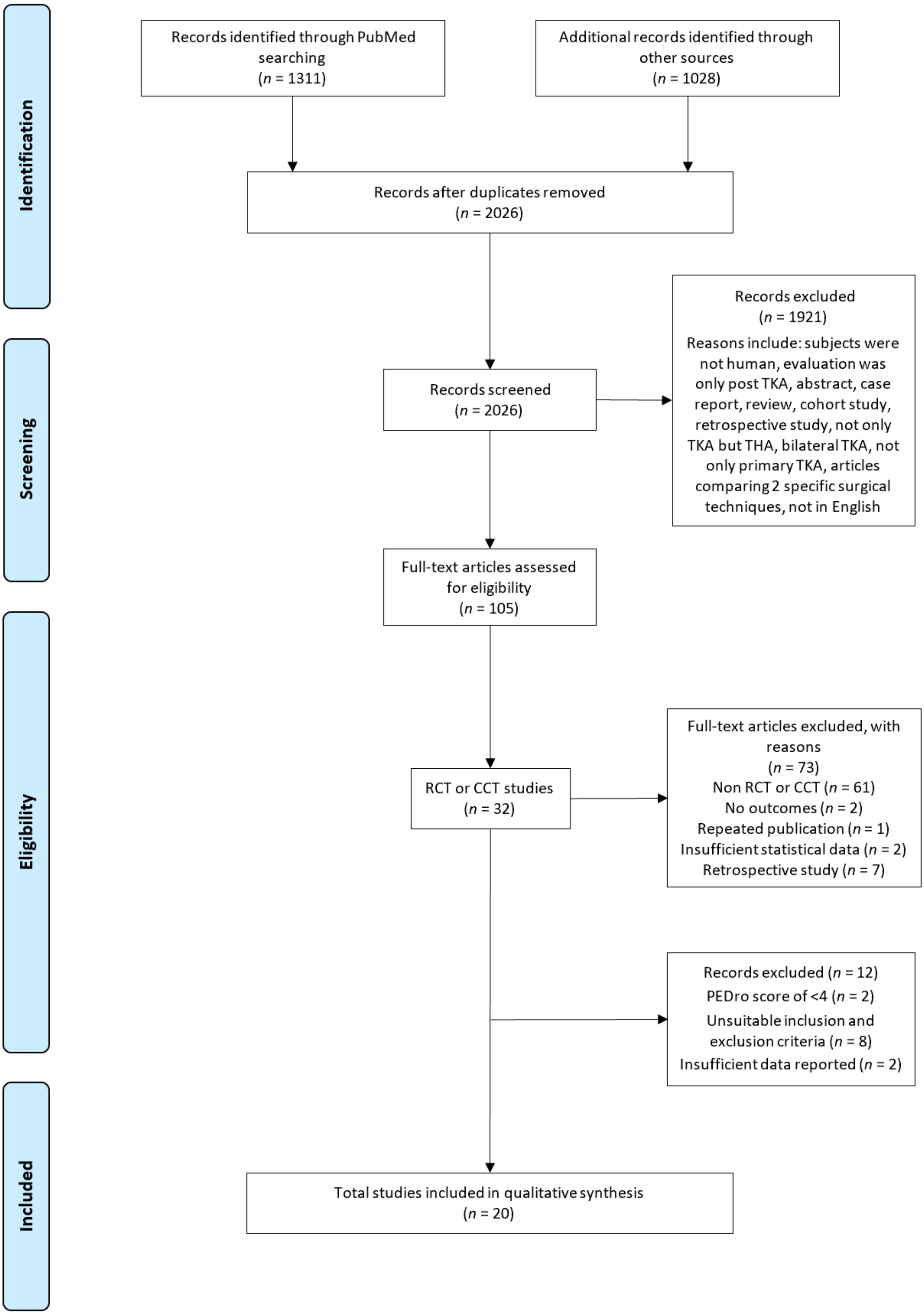

Figure 1. PRISMA flowchart diagram of the search process. RCT, randomized controlled trial; CCT, clinical controlled trial; TKA, total knee arthroplasty.

Table 1. Articles included in the review.

Author	Year of Publication	Country	Journal §	Sample Size	Number of Centers, Study Design	Primary Outcome	PEDro Score˥
Winters et al. [33]	2014	USA	Knee	nE = 35, nC = 23	1, CCT	Quadriceps and hamstrings isometric strength assessment	4
Smith et al. [34]	2014	USA	J Arthroplasty	nE = 13, nC = 11	1, CCT	Quadriceps muscle strength by isokinetic dynamometer	5
Bade et al. [35]	2014	USA	Am J Phys Med Rehabil	nE = 34, nC = 30	1, RCT	Knee ROM	5
Abdel et al. [36]	2014	USA	Clin Orthop Relat Res	nE = 20, nC = 20	1, RCT	KSS, KOOS, SF-12	7
Jenkins et al. [37]	2014	USA	J Bone Joint Surg Am	nE = 60, nC = 60	1, RCT	Quadriceps isometric strength assessment	7
Thomas et al. [38]	2014	USA	Knee	nE = 10, nC = 10	1, CCT	Quadriceps and hamstrings isometric strength assessment	5
Huber et al. [39]	2015	Switzerland	BMC Musculoskelet Disord	nE = 22, nC = 23	1, RCT	CST	7
Calatayud et al. [40]	2017	Spain	Knee Surg Sports Traumatol Arthrosc	nE = 25, nC = 25	1, RCT	WOMAC	7
Bistolfi et al. [41]	2017	Italy	Knee Surgery Sports Traummatol Arthrosc	nE = 23, nC = 44	1, CCT	HDRS	4
Hadlandsmyth et al. [42]	2017	USA	Psychol Health Med	nE = 173, nC = 173	1, RCT	NRS pain	4
Cooper et al. [43]	2017	USA	J Orthop Res	nE = 62, nC = 62	1, CCT	KOOS, gait velocity and physical activity level using accelerometry	5
Bonnefoy-Mazure et al. [44]	2017	Switzerland	J Arthroplasty	nE = 79, nC = 32	1, CCT	Gait velocity and knee ROM	5
Loyd et al. [45]	2017	USA	Phys. Ther	nE = 84, nC = 78	4, RCT	Strength testing by isokinetic dynamometer	7
Lin et al. [46]	2018	Taiwan	J. Clin. Nurs	nE = 100, nC = 100	1, RCT	KOOS	7
Husby et al. [47]	2018	Norway	Eur J Phys Rehabil Med	nE = 21, nC = 20	1, RCT	Quadriceps and hamstring muscle strength	6
Paravlic et al. [48]	2019	Slovenia	PLoS ONE	nE = 17, nC = 17	1, RCT	Quadriceps and hamstring muscle strength	7
Indelli et al. [49]	2019	USA	Knee Surg Sports Traumatol Arthrosc	nE = 50, nC = 50	1, RCT	ROM, KSS, OKS	6
Jiang et al. [50]	2019	China	Orthop Surg	nE = 109, nC = 147	1, CCT	NRS pain, KSS, ROM	5
Liljensøe et al. [51]	2019	Denmark	Scand J. Surg	nE = 38, nC = 38	1, RCT	SF-36	7
Skoffer et al. [52]	2019	Danmark	Clin Rehabil	nE = 30, nC = 29	1, CCT	5STS test	8

E = experimental; C = control; RCT = randomized controlled trial; CCT = clinical controlled trial §. See Table 2 for additional abbreviations. Short name of the journal in which the article was published. ˥ PEDro score: sum of PEDro (Physiotherapy Evidence Database) scale item scores (see Table A2 for details on scores).

Table 2. Summary of knee osteoarthritis (OA) outcomes used in included articles.

Abbreviation	Full Name	Used as Primary or Secondary Outcomes	Number of Times Used
**Questionnaires**			
KOOS	Knee injury and Osteoarthritis Outcomes Score	Primary	6
WOMAC	Western Ontario and McMaster Universities Osteoarthritis index	Primary	2
KSS	New Knee Society Score	Primary	4
OKS	Oxford Knee Score	Primary	1
HSS	Hospital for Special Surgery	Secondary	1
HDRS	Hamilton Depression Rating Scale	Primary and secondary	1
STAI	State-Trait Anxiety Index	Secondary	2
GDS	Geriatric Depression Scale	Secondary	2
HADS	Hospital Anxiety and Depression Scale	Secondary	1
SF-36	MOS Short Form 36	Primary and secondary	6
SF-12	MOS Short Form 12	Secondary	2
PCS	Pain Catastrophizing Score	Secondary	2
NRS pain	Pain intensity by Numeric Rating Scale	Primary and secondary	8
**Physical tests**			
Knee ROM	Knee Range Of Motion	Primary and secondary	8
ASLR	Active Straight Leg Raise	Secondary	1
Strength	Strength testing of knee flexor/extensor by isometric or isokinetic dynamometer	Primary and secondary	8
EMG	Quadriceps/hamstrings co-activation and on/off timing using electromyography	Secondary	1
PPT	Pressure Pain Threshold	Secondary	1
Gait velocity	Gait Velocity (self-paced)	Primary and secondary	4
10MWT	Maximum walking speed/10-m walk test (fast-paced)	Secondary	1
6MWT	Six-Minute Walk test	Secondary	4
Actimetry	Physical activity level with average steps/day using accelerometry	Primary and secondary	2
5STS test	Five Times Sit-To-Stand Test	Primary and secondary	4
TUG	Timed Up and Go Test	Primary and secondary	6
SCT	Stair Climbing Time	Secondary	2

Table 3. Description of patient-reported outcome measures used for patient with knee OA undergoing total knee arthroplasty.

Tool (References)	Target Population #	Domains ˥	No. of Scales ˩	No. of items	No. of Response Options	Range of Scores	Time to Administer (min)	Mode of Administration	Cultural/Adaptation ǂ	Copyright
**Condition-specific**										
KOOS [53–57]	Knee OA	Pain, other disease-specific symptoms, ADL function, sport and recreation, function, knee related QoL	5	42	5	P: 0–36 Sy: 0–28 A: 0–68 SP: 0–16 Q: 0–16	10	Self-administered	Many languages	No
WOMAC [53,58–62]	Knee OA	Pain, physical function, stiffness	3	24	5	P: 0–20 S: 0–8 PF: 0–68	5-10	Self-administered	Many languages	No
KSS [63,64]	Knee OA	Pain, expectation, satisfaction, physical function	4	34	Varies	O: 0–100 Sa: 0–40 Ex: 0–15 F: 0–100	15	Interview based and examination	English French German Chinese Portuguese Dutch Turkish	Yes
OKS [65–68]	Knee OA	Pain and physical function	1	12	5	12–60	10	Self-administered	Many languages	Yes
HSS [69,70]	TKA	Expectation	1	19	5	0-100	5-10	Self-administered	English French	No
**Patient-specific**										
HDRS	Depressed patients	Depression	1	17	Varies	0–53	20–30	Interview based	Many languages	Yes
STAI [71]	General population	Anxiety	2	40	4	S-A: 20–80 T-A: 20–80	10	Self-administered	Many languages	Yes
GDS [72]	Elderly	Depression	1	30	2	0–30	5–10	Self-administered	Many languages	No
HADS [71–75]	General population	Anxiety and depression	2	14	4	A: 0–21 D: 0–21	5–10	Self-administered	Many languages	Yes
**Generic**										
SF-36 [20,21,60,61,76–78]	General population	Pain, physical/mental/social function, general health	8	36	Varies	0–100 PCS: 0–100 MCS: 0–100	10	Self-administered	Many languages	No
SF-12 [21,61,77,79,80]	General population	Pain, physical/mental/social function, general health	2	12	Varies	0–100 PCS: 0–100 MCS: 0–100	5	Self-administered	Many languages	No
PCS [81]	General population	Pain catastrophizing	1	13	5	0–52 R: 0–16 M: 0–12 H: 0–24	5	Self-administered	English French	Yes
NRS pain [82]	General population	Pain severity	1	1	11	0–10	1	Self-administered	No translation needed	No

OA = osteoarthritis; TKA = total knee arthroplasty; ADL = activities of daily living; QoL = quality of life; P = pain subscale; Sy = symptoms subscale; A = activity limitations of daily living; SP = activity limitations for sport and recreation; Q = quality of life (knee related); S = stiffness subscale; PF = physical function subscale; O = objective subscale; Sa = satisfaction subscale; Ex = expectation subscale; F = functional activity subscale; S–A: the state anxiety scale; T–A: the trait anxiety scale; A: anxiety ; D: depression; PCS = physical component summary; MCS = mental component summary; R = rumination; M = magnification ; H = helplessness; see Table 2 for additional abbreviations.# Population for which the questionnaire has been developed; ˥ Domains: domain(s) explored by the tool; ˩ Scales: a subscore within a tool; ǂ non-exhaustive list, see Table A3 for more details.

3.4. Risk of Bias Within Studies

Eleven trials were considered high quality (PEDro score >5/10), with a mean score of 5.9/10 across all trials (Table 1 and Table A2). The total PEDro scores were 8 for 1 trial [52], 7 for 8 trials [1,20,43,48,57,58,60,73], 6 for 2 trials [44,45], 5 for 6 trials [11,24,49,82–84] and 4 for 3 trials [40,85,86]. The items of the PEDro scale the most frequently found were eligibility criteria, outcome obtained in more than 85% of participants, the use of similar groups at baseline, measurements of variability for at least one key outcome, and between-group comparisons, which were evident in almost all reports. None of the trials reported blinding of participants or therapists nor assessors, which is expected, given that these items are the most difficult to adhere to in trials of non-pharmacological interventions involving exercise. Nineteen trials reported an intention-to-treat analysis, 9 used allocation concealment and 10 used random allocation.

3.5. Psychometric Qualities of Tools

3.5.1. Content Validity

Content validity was assessed for 9 (69%) of the 13 tools (Figure 2 and Table A4). The Knee Injury and Osteoarthritis Outcome Score (KOOS) [53], OKS [29,41], State-Trait Anxiety Index (STAI) [71], Geriatric Depression Scale (GDS) [72] and Hospital Anxiety and Depression Scale (HADS) [10,17,51,81,87] had positive ratings based on several studies. The WOMAC [8,18,33,62,78], New Knee Society Score (KSS) [28,69], SF-36 [7,8,26,33,74,88], SF-12 [7,27,33,37,88] and Pain Catastrophizing Score (PCS) [81] had indeterminate (doubtful) ratings regarding the lack of clear and complete documentation of the item selection process.

3.5.2. Internal Consistency

Both factor analysis (or a similar method, e.g., principal component analysis) and calculation of Cronbach α were performed for only 6 (46%) tools (or particular subscales only, e.g., pain), namely, WOMAC [8,18,62,78,89], KOOS [54], KSS [28,69], OKS [29,41], STAI [71] and GDS [72]. For most of the remaining studies, the Cronbach α was determined, and studies involving the SF-36 [33,34], SF-12 [7,27,37,88] and PCS [81] showed values of ≥0.7 for all subscales. Although the HADS [10,17,51,81,87] had a positive rating based on several studies, it may show reduced validity in some populations (e.g., older people).

3.5.3. Construct Validity

Construct validity was assessed in all tools. The KOOS [36,39,72,77,78], WOMAC [8,18,33,62,78,89], OKS [29,41], GDS [72], HADS [10,17,51,81,87], SF-36 [7,8,26,33,34,74,88], SF-12 [7,27,33,37,88], and pain by a numeric rating scale (NRS pain) [82] had positive ratings based on some studies. The KSS [28,69] and PCS [81] received indeterminate ratings mostly due to the lack of pre-defined hypotheses and confirmation of less than 75% of the hypotheses. Finally, the HSS [50,68] and STAI [71] obtained negative assessments.

3.5.4. Floor/Ceiling Effects

Floor and ceiling effects were evaluated in only 7 (54%) instruments. Based on more than one study, the KOOS [36,39,72,77,78], WOMAC [8,18,33,62,78,89], OKS [29,41,90], SF-36 [7,8,26,33,34,74,88], and SF-12 [7,27,33,37,88] had positive ratings. The HADS [10,17,51,81,87] and HSS [50,68] had indeterminate ratings in nearly all studies because some subscales did not meet the 15% cut-off point.

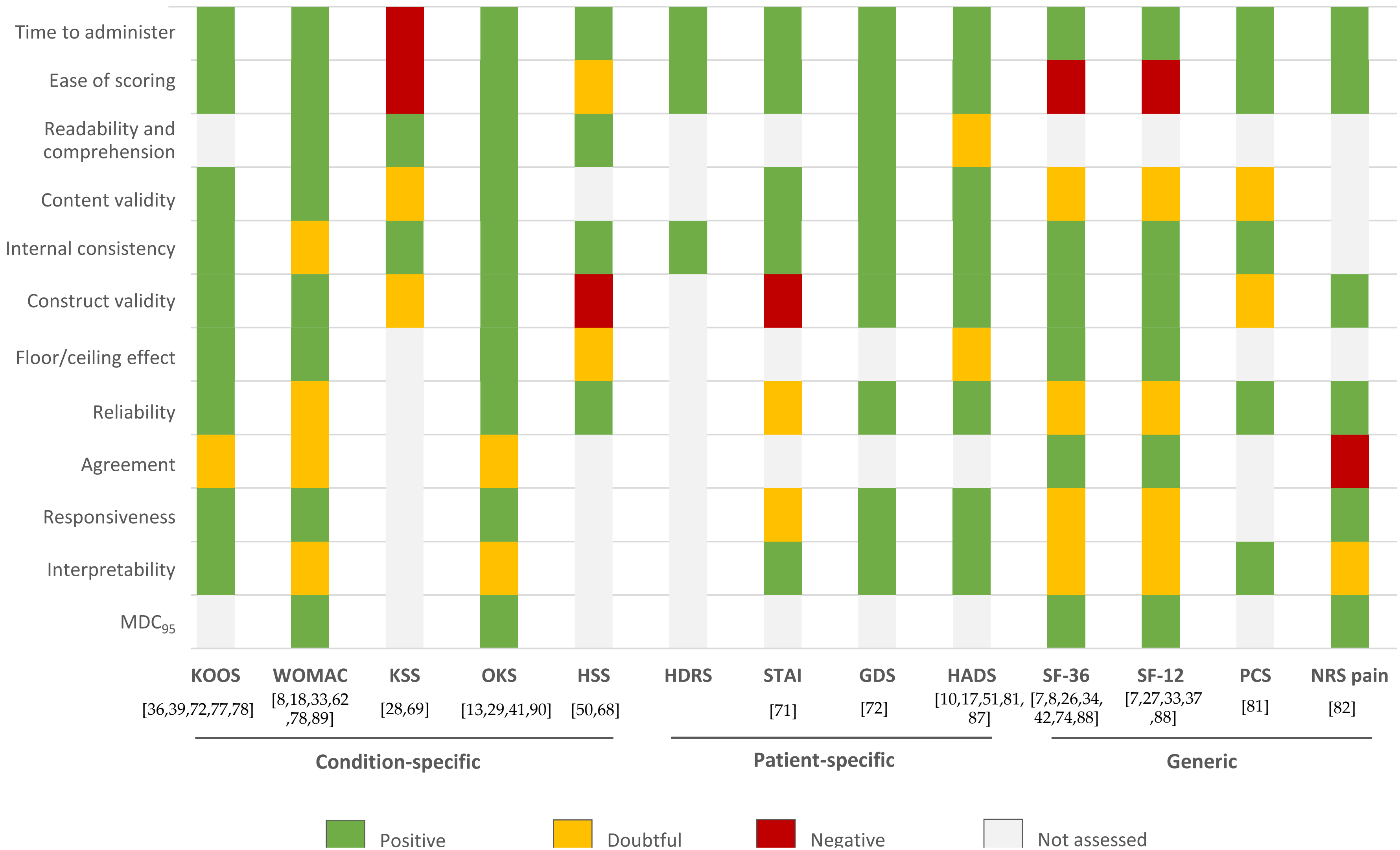

Figure 2. Summary of the quality assessment of patient -reported outcome measures included. MDC_{95} = minimal detectable chance at the 95% confidence level; see Table 2 for additional abbreviations.

3.5.5. Reliability

Reliability parameters were reported in 11 (85%) instruments. The KOOS [54], OKS [13,29], HSS [50,68], GDS [72], HADS [10,17,51,81,87], PCS [81] and NRS pain [82] had positive ratings in terms of the intra-class correlation coefficient (ICC), generally ranging from 0.7 to 0.9, based on some studies. However, the WOMAC [62], SF-36 [61], and SF-12 [61] had indeterminate ratings based on several studies. The STAI [71] also had indeterminate ratings because of poor reliability for the S-Anxiety subscale. Indeterminate ratings were attributed to coefficients <0.7 for a number of subscales, sample sizes <50, and uncertainty of methods used, such as Pearson's correlation (Table S1).

3.5.6. Agreement

Agreement was evaluated in 5 (38%) tools; namely, KOOS [39,72,78], WOMAC [62], SF-36 [7,8,26,33,34,74,88], SF-12 [7,27,33,37,88] and NRS pain [82]. Only the SF-36 and SF-12 had positive ratings. The KOOS [39,72,78] had indeterminate ratings based on several studies, primarily because of small sample sizes, as did the WOMAC [62]. For KOOS [54] and WOMAC, the calculated standard error of the mean (SEM) and minimal detectable change (MDC) were compared by 0.5 standard deviation (SD), because the minimal important change (MIC) was not defined and these values were less than the estimated MIC.

3.5.7. Responsiveness

Responsiveness was examined in almost all tools by various methods. With the definition of responsiveness used in this study, the KOOS [39,72,78], OKS [13,29,41,90], GDS [72], HADS [10,17,51,81,87] and NRS pain [82] had positive ratings for responsiveness to change. In TKA patients, at 6 months, the MIC scores for improvement in pain and physical function for the WOMAC were approximately 23 and 19, respectively, which were higher than the MDC [78]. For the NRS pain, the MIC was less than the MDC [82]. The remaining instruments had indeterminate ratings because only distribution-based methods were used, external clinical criteria or a control ("stable") population to determine whether change had indeed occurred were lacking, and the MIC was not defined.

3.5.8. Interpretability

For 5 (38%) instruments, at least 2 types of information were presented to aid interpretability. Interpretability was rated positive for the KOOS [36,39,72,77,78], STAI [71], GDS [72], HADS [10,17,51,81,87] and PCS [81]. The remaining tools had doubtful or no ratings mostly because of presentation of less than 2 types of information.

3.5.9. Practical Burden

Practical burden on the patient (time to complete tool, ease of scoring) was assessed for almost all instruments. Only the KSS, HSS, SF-36 and SF-12 had doubtful or negative ratings, which were mainly related to some scoring difficulty.

3.5.10. Cultural Adaptation

Transcultural adaptation was not rated precisely because of the great subjectivity and frequently because of lack of clarity in the cross-cultural adaptation process. Table 3 and Table A3 show the cultural translations/adaptations of each tool.

3.6. Psychometric Qualities of Physical Tests

3.6.1. Reliability

Reliability parameters were reported for almost all physical tests. The strength testing of knee flexor/extensor by isometric or isokinetic dynamometer (strength) [87], 6-min Walk Test (6MWT) [9,53,91], Five Times Sit-To-Stand Test (5STS test) [42,63,92], Timed Up and Go Test (TUG)

[2,42,53,91,93] and Stair Climbing Time (SCT) [6,32,94] test had positive ratings for both intra- and inter-tester reliability. The Pressure Pain Threshold (PPT) [86] had a positive rating for intra-tester reliability, but inter-tester reliability was not assessed. The other physical tests had doubtful reliability.

3.6.2. Responsiveness

Only strength [87], PPT [86], 6MWT [9,53,91] and TUG [2,42,53,91,93] tools had a positive rating for responsiveness. The others were doubtful for gait velocity at self-paced and 10-m Walk Test (10MWT) at a fast pace or undetermined for the remaining tests.

3.6.3. Interpretability

For 6 (50%) tests, interpretability was rated positive for the knee range of motion [47,54], strength [87], PPT [86], 6MWT [9,53,91], 5STS [42,63,92] and TUG [2,42,53,91,93] tools. The remaining tools had doubtful or no ratings mostly due to lack of MDC.

3.6.4. Practical Burden

Practical burden on the patient (time to administer, ease of scoring) was assessed for almost all instruments. Only strength had negative ratings related to time to administer. Physical activity level with average steps/day measured by accelerometry (actimetry) also had a negative rating related to ease of scoring, with some signal processing difficulty.

3.7. Overall Score

Among the condition-specific PROMs, the OKS, KOOS and WOMAC had the highest overall scores with 10, 9 and 8 positive ratings respectively over the 12 criteria investigated. Concerning generic tools, the SF-36 and SF-12 obtained the highest overall score, with 6 positive ratings. For patient-specific tools, the GDS and HADS seem appropriate, with an overall positive rating of 9 and 8, respectively (Table 3 and Figure 2).

Regarding physical tests, some tests appeared to be quite robust in terms of psychometric properties—strength, PPT, 6MWT, 5STS, TUG tools—with an overall score ≥6/7. However, some tests lacked clinimetric studies: The Active Straight Leg Raise (ASLR), gait velocity, 10MWT, electromyography and actimetry, with an overall score ≤2 (Figure 3 and Table A5).

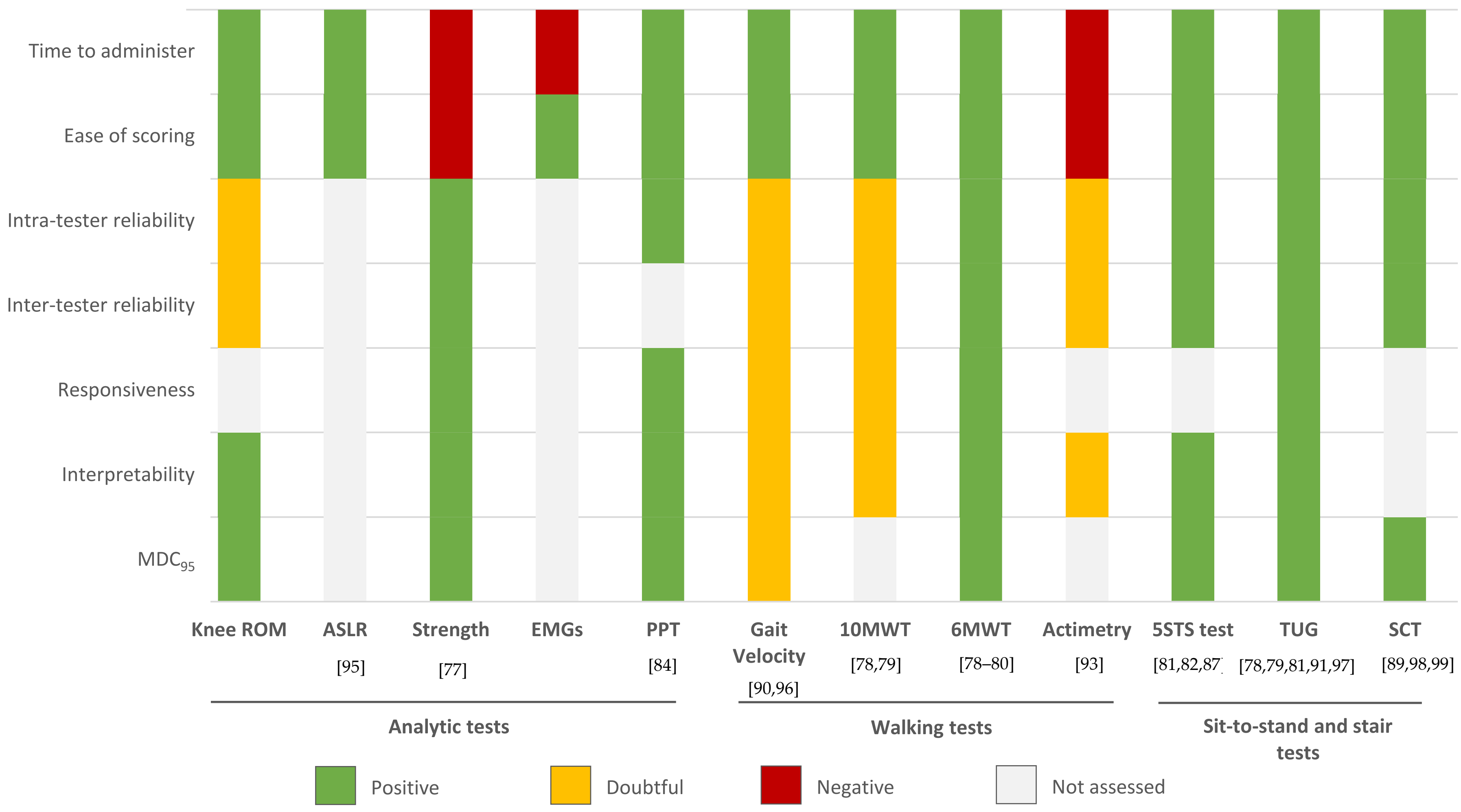

Figure 3. Summary of the quality assessment of the included physical tests. MDC_{95} = minimal detectable chance at the 95% confidence level; see Table 2 for additional abbreviations.

4. Discussion

In this study, we examined and compared the quality of the measurement properties of outcome measures (patient-reported and for the first time, physical tests) assessing rehabilitation outcomes for patients with knee OA undergoing TKA. There are 3 main findings in this review. First, a wide variety of PROMs are applied to measure outcomes in rehabilitation after knee arthroplasty, but only 3 (KOOS, WOMAC and OKS) have undergone an extensive validation process in knee OA before and/or after TKA. Second, important measurement attributes for evaluative instruments such as reproducibility, responsiveness to clinical change and definition of the MIC are still scarcely evaluated. Third, some physical tests were well evaluated (TUG, 5STS test, 6MWT, PPT) but others not at all (actimetry, electromyography, ASLR).

Of the 13 tools applied in knee arthroplasty rehabilitation, the KOOS, WOMAC and OKS have been completely studied for their measurement properties, including content validity, reliability, construct validity, responsiveness, and floor and ceiling effects. The GDS seems valid in younger populations but in view of the mean age of TKA patients may not be the best assessment and the HADS may be preferred. The Hamilton Depression Rating Scale (HDRS) is frequently used as a primary or secondary outcome, but no studies have been carried out in this type of population; nevertheless its validity has been assessed in a depressive population [96]. In the same way, for the ASLR, although frequently used, only one study used it in this type of population [93] but not really in terms of its psychometric properties. Nevertheless, clinimetric properties of the ASLR have been widely assessed in chronic low back pain [100].

Another key finding from this study is the persistent lack of essential requisites for evaluative instruments. Because evaluative PROMs are used to assess change in patients over time, these measures need to have high responsiveness and high reproducibility [101]. These instruments must be able to identify a change if it occurred, and in this case be able to establish that it is beyond the range of measurement error. Therefore, the agreement parameter is vital (and preferred over the reliability parameter) because this concerns the absolute measurement error. The measurement error is an essential data to distinguish whether the change measured is relevant or not. The MDC can be estimated from the measurement error, and can be compared to the MIC. Knowing the MDC, the amount of measurement error as well as how these relate to the MIC provides insight into the meaning of values changes from the instrument [32].

In this review, as in the study of Veenhof et al. [20], estimates of agreement were reported less frequently than were estimates of reliability. Of the 13 instruments, 6 (46%) reported a positively rated reliability parameter and only 2 (15%) presented an agreement parameter. Researchers tend to use more reliability parameters than agreement parameters [101]. Agreement parameters is a psychometric property tend to be neglected in clinimetric studies in the medical sciences [101]. The other issue lay in determining the responsiveness of the instruments for which a definition of MDC is missing. According to Alviar et al. [21], "most studies examined responsiveness to clinical change by estimating effect sizes, standardized response means, and the t statistic, which could be affected by sample size and sample variation". In this review, as in Alviar et al. ten years ago, only a few studies defined the MDC to allow for meaningful interpretation of the obtained scores. Defining what constitutes a clinically meaningful change should remain a priority for future clinimetric studies.

Although the HDRS has been widely used to evaluate depression, to our knowledge no clinimetric study has been done in OA patients. Moreover, the criticism regarding its reliability has been growing the last few years. A recent review of Bagby et al. [96] showed that the HDRS is ''… psychometrically flawed…''and the authors suggested its revision.

Some physical tests were well evaluated, but others not at all. Actimetry is a recent technology in full expansion, having for a main interest allowing an evaluation of the functional abilities of the patient in everyday life. However, we lack consensus on the measurement method, signal processing and results interpretation (number of steps, activity counts, duration of activity at different intensity levels). Mainly, the lack of consensus is from the signal processing and extracted data but not from interpretation of these data for which there is a known threshold of "normality". Studies of this

subject seem necessary. Also, the tests presented here (with the exception of strength, EMG and actimetry) are simple to implement in everyday clinical practice (see necessary equipment in Table A1 and Table A5). However, although most have psychometric properties clearly evaluated in our population, the rules of good practices must be respected when administering these performance-based tests (see Table A1).

This study has several limitations. Regarding the large number of studies of TKA patients, only instruments used in CCTs or RCTs were included, so pertinent tools might have been missed. The definitions approaches and methods in assessing the attributes varied among studies. We lack a gold standard to assess the measurement properties of PROMs [88]. The review included only studies in which measurement properties were assessed with classical response theory, and therefore recent studies using relatively newer approaches (e.g., IRT) might have been missed. The IRT method is progressively becoming a prominent tool in rehabilitation research since it facilitates the evaluation of the quality of psychometric properties of some instruments. However, we still lack explicit criteria for quality evaluation of the methods and results of studies using IRT models. The unfavorable or indeterminate ratings a tool received could be due to flaws in study methods, and not necessarily deficiency of the tool per se. In addition, because some tools have been extensively studied (e.g., WOMAC, SF-36), they had varied ratings per measurement attribute as compared with others with only one or a few clinimetric studies but positive ratings for the attributes. Negative results in clinimetric studies obtained and not published (publication bias) is another limitation, which might have precluded the inclusion of these studies.

5. Conclusions

We compared the measurement attributes of the various outcomes applied in studies of rehabilitation after TKA with a view to facilitating the choice of the most appropriate instruments for studies of patients with knee OA who are undergoing TKA. Physical tests were reviewed for the first time in TKA population. Our analysis suggests that strength, PPT, 6MWT, 5STS and TUG tools are notable; the 6MWT, TUG being however the most extensively validated and therefore possibly the most appropriate to use. Overall, regarding PROMs our findings corroborate results from previous studies suggesting that the KOOS, WOMAC, OKS, HADS and SF-36 are the most comprehensively tested tools in this population and are worth considering. Nevertheless, as already demonstrated more rigorous evaluations in key areas, such as reproducibility, responsiveness to clinical change, and MIC, are still needed to make stronger recommendations. By differentiating the assessment field of each tool, we may potentially recommend the KOOS for the most relevant as à condition specific questionnaire; the HADS as a patient specific and finally the SF-36 as a generic one. Nevertheless, other promising assessments (e.g., actimetry) lack validation and require rigorous studies to be used as a core set of outcomes in future studies. This review could serve as a basis for future studies, being a guarantee of quality.

Supplementary Materials: The following are available online at www.mdpi.com/xxx/s1 and detail the Experimental Section, Research algorithm S1: Search terms used in databases, Table S1: Checklist for rating the psychometric quality of self-assessment tools, Table S2: Checklist for rating the psychometric quality of physical tools.

Author Contributions: V.R.: conceptualization, investigation, analysis and interpretation of the data and writing the original draft. A.V.: conceptualization, investigation, analysis of the data and editing the manuscript. B.P.: conceptualization, methodology, editing the manuscript. S.B.: validation, review and editing the manuscript, F.C.: conceptualization, validation, review and editing the manuscript, E.C.: conceptualization, project administration, validation, review and editing the manuscript.

Funding: This research received no external funding

Conflicts of Interest: The authors declare no conflict of interest.

Appendix

Table A1. Description of performance-based tests of physical function.

Performance-Based Tests	Equipment/Space	Measure	Description/Instructions
**Walking tests**			
10MWT	Stopwatch 10 m (33 ft) marked walkway with an additional 2 m at each end for acceleration and deceleration.	Speed (m/s)	Walk as quickly but as safely as possible to a mark 10 m away starting off launched. Regular walking aid is allowed.
6MWT	Timer/stopwatch Flat, hard-surfaced indoor walkway of 30 m marked with 1 m intervals. Chair or stool for resting	Distance (m)	The maximum distance that can be walked over a 6-min interval is recorded. Rest periods are allowed but included in the time Standardized encouragement (e.g., “keep going you are doing really well”) can be given at minute intervals. Regular walking aid is allowed. Practice test not needed in most clinical settings but if performed then at least 1 h rest should be allowed before the second test. The greatest distance is then recorded.
**Sit-to-stand tests**			
5STS test	Timer/stopwatch Straight back chair preferably without arms with approximately 43 cm (17 inch) seat height	Time (s)	From the sitting position with arms crossed at chest, stand up (sit down) as fast as possible for a total of five stands (i.e., test ends in standing). Fastest time of two trials is recorded in seconds. Same chair is required for re-testing.
TUG	Stopwatch Standard chair with armrests (approx. 46 cm (18 inch) seat height with 65 cm (26 inch) arm rest height) Marked 3 m (10 ft) walkway with turn point at end	Time (s)	Time to rise from a standard armchair, walk as quickly but as safely as possible distance of 3 m, turn, walk back to the chair and sit down. Usual footwear and regular walking aids allowed and recorded. Fastest of two trials is recorded in seconds. Same chair is needed for re-testing.
**Stair tests**			
SCT	Stopwatch Flight of 12 stairs with 18 cm (7 inch) step height and handrails	Time (s)	Ascend and descend flight of 12 stairs as quickly as safe and comfortable. One handrail allowed but encouraged to only use legs. Total time to ascend and descend steps for one trial is recorded to nearest 100th second.

See Table 2 for abbreviations.

Table A2. PEDro scores for included studies in this review.

Reference	1 *	2	3	4	5	6	7	8	9	10	11	Total (/10)	Quality
Winters et al., 2014 [33]	Y	0	0	0	0	0	0	1	1	1	1	4	Moderate
Smith et al., 2014 [34]	Y	0	0	1	0	0	0	1	1	1	1	5	Moderate
Bade et al., 2014 [35]	Y	0	0	1	0	0	0	1	1	1	1	5	Moderate
Abdel et al., 2014 [36]	Y	1	1	1	0	0	0	1	1	1	1	7	High
Jenkins et al., 2014 [37]	Y	1	1	1	0	0	0	1	1	1	1	7	High
Thomas et al., 2014 [38]	Y	0	0	1	0	0	0	1	1	1	1	5	Moderate
Huber et al., 2015 [39]	Y	1	1	1	0	0	0	1	1	1	1	7	High
Calatayud et al., 2017 [40]	Y	1	1	1	0	0	0	1	1	1	1	7	High
Bistolfi et al., 2017 [41]	Y	0	0	1	0	0	0	1	0	1	1	4	Moderate
Hadlandsmyth et al., 2017 [42]	Y	0	0	0	0	0	0	1	1	1	1	4	Moderate
Cooper et al., 2017 [43]	Y	0	0	1	0	0	0	1	1	1	1	5	Moderate
Bonnefoy-Mazure et al., 2017 [44]	Y	0	0	1	0	0	0	1	1	1	1	5	Moderate
Loyd et al., 2017 [45]	Y	1	1	1	0	0	0	1	1	1	1	7	High
Lin et al., 2018. [46]	Y	1	1	1	0	0	0	1	1	1	1	7	High
Husby et al., 2018 [47]	Y	1	0	1	0	0	0	1	1	1	1	6	High
Paravlic et al., 2019 [48]	Y	1	1	1	0	0	0	1	1	1	1	7	High
Indelli et al., 2019 [49]	Y	0	0	1	0	0	1	1	1	1	1	6	High
Jiang et al., 2019 [50]	Y	0	0	1	0	0	0	1	1	1	1	5	Moderate
Liljensøe et al., 2019 [51]	Y	1	1	1	1	0	0	0	1	1	1	7	High
Skoffer et al., 2019 [52]	Y	1	1	1	1	0	0	1	1	1	1	8	High

* Not included in total score; Y = yes; N = No; 1 = eligibility criteria; 2 = random allocation; 3 = concealed allocation; 4 = similarity groups at baseline; 5 = blinding subjects; 6 = blinding therapists; 7 = blinding assessors; 8 = outcome obtained in >85% of the subjects; 9 = intention to treat analysis; 10 = between-group statistical comparison; 11 = point estimates and measures of variability.

Table A3. Cultural translation/adaptation for each tool.

Tool	Translations/Adaptations
**Condition-specific**	
KOOS	Available in English. The KOOS has been translated into Arabic, Austria-German, Bengali, Czech, Chinese, Croatian, Dutch, Danish, Estonian, Finnish, Filipino, French, Greek, Icelandic, Italian, Japanese, Korean, Latvian, Lithuanian, Malay, Norwegian, Persian, Portuguese, Polish, Romanian, Russian, Slovakian, Singapore English, Slovenian, Spanish, Swedish, Thai, Turkish, Ukrainian, Vietnamese, Welsh, and Zulu.
WOMAC	Available in English. The WOMAC has been translated into Arabic, German, French, Hebrew, Italian, Japanese, Singapore, Spanish, Korean, and Swedish.
KSS	Available in English. The KSS has been translated into French, German, Chinese, Portuguese, Dutch, and Turkish.
OKS	Available in English. The KSS has been translated into French, German, Chinese, Portuguese, Dutch, and Turkish. French, Portuguese, German, Danish, Thai, Estonian, Finnish, Greek, Indonesian, Japanese, Chinese, Russian, Arabic, Serbian, Swedish, Italian, Turkish, Malay, Welsh, Polish, Korean, Spanish.
HSS	Available in English and French
Patient-specific	
HDRS	Available in English. The HDRS has been translated into many languages such as Arabic, Bulgarian, Croatian, French, German, Georgian, Hebrew, Italian, Japanese, Russian, Spanish,
STAI	Available in English. The STAI has been translated into more than 48 languages
GDS	Available in English. The GDS has been translated into Arabic, Chinese, Creole, Danish, Dutch, Farsi, French, French Canadian, German, Greek, Hebrew, Hindi, Hungarian, Icelandic, Italian, Japanese, Korean, Lithuanian, Malay, Maltese, Norwegian, Portuguese, Romanian, Russian, Serbian, Spanish, Swedish, Thai, Turkish, Vietnamese, and Yiddish.
HADS	Available in English, as well as all other languages of Western Europe and many of Eastern Europe and Scandinavia, along with some African and Far East languages, including Arabic, Chinese, Danish, Dutch, Finnish, French, German, Hebrew, Hungarian, Italian, Japanese, Korean, Norwegian, Portuguese, Spanish, Swedish, Thai, and Urdu.
**Generic**	
SF-36	Available in English. The SF-36 has been translated into more than 161 languages (Chinese, Dutch, Danish, French, Iranian, Italian, Japanese, Kiswahili, Norwegian, Portuguese, Spanish, and Swedish)
SF-12	Available in English. The SF-36 has been translated into more than 141 languages.
PCS	Available in English and French
NRS pain	No translation needed.

See Table 2 for abbreviations.

Table A4. Summary of the quality assessment of the patient-reported outcome measures included.

Tool (References)	Time to Administer	Ease of Scoring	Readability and Comprehension	Content validity	Internal Consistency	Construct Validity	Floor/Ceiling Effect	Reliability	Agree-ment	Respon-siveness	Interpre*t*-ability	MDC_{95}	Positively Rated Qualities, no
**Condition-specific**													
KOOS [36,39,72,77,78]	+	+		+	+	+	+ §§§	+	ø	+	+		9
WOMAC [8,18,33,62,78,89]	+	+	+	+	ø	+	+ §	ø	ø	+	ø	+ 0.5–1.3pts	8
KSS [28,69]	-	-	+	ø	+ *	ø							2
OKS [13,29,41,90]	+	+	+	+	+	+	+ §§§§	+	ø	+	ø	+ 4.15pts	10
HSS [50,68]	+	ø	+		+	-	ø	+					3
**Patient-specific**													
HDRS													0
STAI [71]	+	+		+	+	-		ø #		ø J	+		5
GDS [72]	+	+	+	+	+	+		+		+	+		9
HADS [10,17,51,81,87]	+	+	ø	+	+ **	+	ø	+		+	+		8
**Generic**													
SF-36 [7,8,26,33,34,74,88]	+	-		ø	+	+	+ § and §§	ø	+	ø	ø	+ 2.0–7.8pts	6
SF-12 [7,27,33,37,88]	+	-		ø	+	+	+ § and §§	ø	+	ø	ø	+ 3.8–5.4pts	6
PCS [81]	+	+		ø	+	ø		+			+		5
NRS pain [82]	+	+				+		+	-	+	ø	+ 1.33 pts	6

MDC_{95} = minimal detectable chance at the 95% confidence level; + = positive; - = negative; ø = doubtful; pts = points; see Table 2 for additional abbreviations.* ø for the functional subscale; ** May have reduced validity in some populations (e.g., older people).§ Floor effect; §§ Ceiling effect; §§§ Floor effect for sports and recreation subscale; §§§§floor and ceiling effect for some individual items but not at the scale level. # - for S-Anxiety. J S-Anxiety more responsive to change than T-Anxiety.

Table A5. Summary of the quality assessment of the included physical tests.

Physical Test (References)	Domains˥	Necessary Equipment ǂ	Time to Administer (min)	Ease of Scoring	Intra-Tester Reliability	Inter-Tester Reliability	Respon-Siveness	Interpret-Ability	MDC_{95}	Positively Rated Qualities, no
**Analytic tests**										
Knee ROM [102,90]	Articular limitation	Goniometer	+ < 5	+	ø	ø		+	+ 6.6–10 °	4
ASLR [93]	Quadriceps strength	Goniometer or measuring tape	+ < 5	+						2
Strength [87]	Lower extremity strength	Isometric or isokinetic dynamometer	−15–30	+	+	+	+	+	+ 5.1–9.3% *	6
EMGs	Neuromotor activation	Surface electromyography	−15–30	-						0
PPT [86]	Knee pain measure	Handheld pressure algometer	+ < 5	+	+		+	+	+ 1.19–1.26 lb	6
**Performance-based tests**										
**Walking tests**										
Gait velocity [103,104]	Patients' functional mobility	Stopwatch, 30 m hallway	+ < 5	+	ø	ø	ø	ø	ø	2
10MWT [91,97]	Patients' functional mobility	Stopwatch, 15m hallway	+ < 5	+	ø	ø	ø	ø		2
6MWT [89,91,97]	Patients' functional mobility	30 m course marked off in meters, stopwatch	+ 10	+	+	+	+	+	+ 61.34 m	6
Actimetry [105]	Patients' physical activity	Accelerometer	- or NA	-	ø	ø		ø		0
**Sit-to-stand tests**										
5STS test [84,98,99]	Lower extremity strength and balance	Chair, stopwatch	+ < 5	+	+	+		+	+ 2.1–2.38 s	6
TUG [91,92,94,97,98]	Patients' functional mobility	Armchair, stopwatch	+ < 5	+	+	+	+	+	+ 1.10–2.27 s	7
**Stair tests**										
SCT [26,88,95]	Patients' functional mobility	Regular stairwell, stopwatch	+ < 5	+	+	+			+ 3.2–5.49 s	5

OA = osteoarthritis; TKA = total knee arthroplasty; MDC_{95} = minimal detectable chance at the 95% confidence level; + = positive; - = negative; ø = doubtful; NA = not applicable; see Table 2 for additional abbreviations.˥ Domains: domain(s) explored by the test; ǂ see Table A1 for more details about equipment, space and instructions; * Standard error of measurement (SEM).

References

1. Cross, M.; Smith, E.; Hoy, D.; Nolte, S.; Ackerman, I.; Fransen, M.; Bridgett, L.; Williams, S.; Guillemin, F.; Hill, C.L.; et al. The global burden of hip and knee osteoarthritis: Estimates from the global burden of disease 2010 study. *Ann. Rheum. Dis.* **2014**, *73*, 1323–1330, doi:10.1136/annrheumdis-2013-204763.
2. O'Neill, T.W.; McCabe, P.S.; McBeth, J. Update on the epidemiology, risk factors and disease outcomes of osteoarthritis. *Best Pract. Res. Clin. Rheumatol.* **2018**, *32*, 312–326, doi:10.1016/j.berh.2018.10.007.
3. Cleveland, R.J.; Alvarez, C.; Schwartz, T.A.; Losina, E.; Renner, J.B.; Jordan, J.M.; Callahan, L.F. The impact of painful knee osteoarthritis on mortality: A community–based cohort study with over 24 years of follow–up. *Osteoarthr. Cartil.* **2018**, doi:10.1016/j.joca.2018.12.008.
4. Deveza, L.A.; Loeser, R.F. Is osteoarthritis one disease or a collection of many? *Rheumatol. Oxf. Engl.* **2018**, *57*, iv34–iv42, doi:10.1093/rheumatology/kex417.
5. Karsdal, M.A.; Bihlet, A.; Byrjalsen, I.; Alexandersen, P.; Ladel, C.; Michaels, M.; Andersen, J.R.; Riis, B.J.; Kraus, V.; Bay-Jensen, A.C.; et al. OA phenotypes, rather than disease stage, drive structural progression—identification of structural progressors from 2 phase III randomized clinical studies with symptomatic knee OA. *Osteoarthr. Cartil.* **2015**, *23*, 550–558, doi:10.1016/j.joca.2014.12.024.
6. Fernandes, L.; Hagen, K.B.; Bijlsma, J.W.J.; Andreassen, O.; Christensen, P.; Conaghan, P.G.; Doherty, M.; Geenen, R.; Hammond, A.; Kjeken, I.; et al. EULAR recommendations for the non–pharmacological core management of hip and knee osteoarthritis. *Ann. Rheum. Dis.* **2013**, *72*, 1125–1135, doi:10.1136/annrheumdis-2012-202745.
7. Lapane, K.L.; Yang, S.; Driban, J.B.; Liu, S.-H.; Dubé, C.E.; McAlindon, T.E.; Eaton, C.B. Effects of prescription nonsteroidal antiinflammatory drugs on symptoms and disease progression among patients with knee osteoarthritis. *Arthritis Rheumatol. Hoboken NJ* **2015**, *67*, 724–732, doi:10.1002/art.38933.
8. Bannuru, R.R.; Osani, M.C.; Vaysbrot, E.E.; Arden, N.K.; Bennell, K.; Bierma-Zeinstra, S.M.A.; Kraus, V.B.; Lohmander, L.S.; Abbott, J.H.; Bhandari, M.; et al. OARSI guidelines for the non-surgical management of knee, hip, and polyarticular osteoarthritis. *Osteoarthr. Cartil.* **2019**, *27*, 1578–1589, doi:10.1016/j.joca.2019.06.011.
9. Maradit Kremers, H.; Larson, D.R.; Crowson, C.S.; Kremers, W.K.; Washington, R.E.; Steiner, C.A.; Jiranek, W.A.; Berry, D.J. Prevalence of Total Hip and Knee Replacement in the United States. *J. Bone Jt. Surg. Am.* **2015**, *97*, 1386–1397, doi:10.2106/JBJS.N.01141.
10. Colas, S.; Occean, B.-V.; Rudnichi, A.; Dray-Spira, R.; Zureik, M. Étude d'utilisation des prothèses articulaires de genou en France entre 2008 et 2013. *Rev. DÉpidémiologie St. Publique* **2016**, *64*, S23, doi:https://doi.org/10.1016/j.respe.2016.01.073.
11. Bellamy, N. Osteoarthritis clinical trials: Candidate variables and clinimetric properties. *J. Rheumatol.* **1997**, *24*, 768–778.
12. Bellamy, N.; Kirwan, J.; Boers, M.; Brooks, P.; Strand, V.; Tugwell, P.; Altman, R.; Brandt, K.; Dougados, M.; Lequesne, M. Recommendations for a core set of outcome measures for future phase III clinical trials in knee, hip, and hand osteoarthritis. Consensus development at OMERACT III. *J. Rheumatol.* **1997**, *24*, 799–802.
13. Pisoni, C.; Giardini, A.; Majani, G.; Maini, M. International Classification of Functioning, Disability and Health (ICF) core sets for osteoarthritis. A useful tool in the follow-up of patients after joint arthroplasty. *Eur. J. Phys. Rehabil. Med.* **2008**, *44*, 377–385.
14. World Health Organization. Regional Office for Europe Guidelines for the clinical investigation of drugs used in rheumatic diseases: European drug guidelines. *Cph. Eur. Leag. Rheum.* **1985**, 49.
15. Allen, K.D.; Bierma-Zeinstra, S.M.A.; Foster, N.E.; Golightly, Y.M.; Hawker, G. OARSI Clinical Trials Recommendations: Design and conduct of implementation trials of interventions for osteoarthritis. *Osteoarthr. Cartil.* **2015**, *23*, 826–838, doi:10.1016/j.joca.2015.02.772.
16. Lequesne, M.; Brandt, K.; Bellamy, N.; Moskowitz, R.; Menkes, C.J.; Pelletier, J.P.; Altman, R. Guidelines for testing slow acting drugs in osteoarthritis. *J. Rheumatol. Suppl.* **1994**, *41*, 65–71; discussion 72-73.
17. Ramkumar, P.N.; Harris, J.D.; Noble, P.C. Patient–reported outcome measures after total knee arthroplasty: A systematic review. *Bone Jt. Res.* **2015**, *4*, 120–127, doi:10.1302/2046-3758.47.2000380.
18. King, S.W.; Cunningham, C.M.; Royeca, J.M.; Madegowda, R.; Sha, S.; Pandit, H. Patient–reported outcome measures review: Are current outcomes assessment tools sensitive enough to assess the reasons for patients' "unhappiness"? *J. Arthrosc. Jt. Surg.* **2020**, doi:10.1016/j.jajs.2020.05.002.

19. Portney, L.G.; Watkins, M.P. *Foundations of Clinical Research: Applications to Practice*, 3rd ed.; Pearson/Prentice Hall: Upper Saddle River, NJ, USA, 2009.
20. Veenhof, C.; Bijlsma, J.W.J.; van den Ende, C.H.M.; van Dijk, G.M.; Pisters, M.F.; Dekker, J. Psychometric evaluation of osteoarthritis questionnaires: A systematic review of the literature. *Arthritis Rheum.* **2006**, *55*, 480–492, doi:10.1002/art.22001.
21. Alviar, M.J.; Olver, J.; Brand, C.; Tropea, J.; Hale, T.; Pirpiris, M.; Khan, F. Do patient–reported outcome measures in hip and knee arthroplasty rehabilitation have robust measurement attributes? A systematic review. *J. Rehabil. Med.* **2011**, *43*, 572–583, doi:10.2340/16501977-0828.
22. Sun, Y.; Stürmer, T.; Günther, K.P.; Brenner, H. Reliability and validity of clinical outcome measurements of osteoarthritis of the hip and knee—a review of the literature. *Clin. Rheumatol.* **1997**, *16*, 185–198, doi:10.1007/bf02247849.
23. Garratt, A.M.; Brealey, S.; Gillespie, W.J.; DAMASK Trial Team Patient–assessed health instruments for the knee: A structured review. *Rheumatol. Oxf. Engl.* **2004**, *43*, 1414–1423, doi:10.1093/rheumatology/keh362.
24. Stewart, L.A.; Clarke, M.; Rovers, M.; Riley, R.D.; Simmonds, M.; Stewart, G.; Tierney, J.F.; PRISMA–IPD Development Group Preferred Reporting Items for Systematic Review and Meta-Analyses of individual participant data: The PRISMA–IPD Statement. *JAMA* **2015**, *313*, 1657–1665, doi:10.1001/jama.2015.3656.
25. Moseley, A.M.; Herbert, R.D.; Sherrington, C.; Maher, C.G. Evidence for physiotherapy practice: A survey of the Physiotherapy Evidence Database (PEDro). *Aust. J. Physiother.* **2002**, *48*, 43–49, doi:10.1016/s0004-9514(14)60281-6.
26. Almeida, G.J.; Schroeder, C.A.; Gil, A.B.; Fitzgerald, G.K.; Piva, S.R. Interrater reliability and validity of the stair ascend/descend test in subjects with total knee arthroplasty. *Arch. Phys. Med. Rehabil.* **2010**, *91*, 932–938, doi:10.1016/j.apmr.2010.02.003.
27. Bot, S.; Terwee, C.; van der Windt, D.; Bouter, L.; Dekker, J.; De Vet, H. Psychometric evaluation of self–report questionnaires: The development of a checklist. *Second Workshop Res. Methodol.* **2003**.
28. Lohr, K.N.; Aaronson, N.K.; Alonso, J.; Burnam, M.A.; Patrick, D.L.; Perrin, E.B.; Roberts, J.S. Evaluating quality–of–life and health status instruments: Development of scientific review criteria. *Clin. Ther.* **1996**, *18*, 979–992, doi:10.1016/s0149-2918(96)80054-3.
29. Bombardier, C.; Tugwell, P. Methodological considerations in functional assessment. *J. Rheumatol. Suppl.* **1987**, *14*, 6–10.
30. Mokkink, L.B.; Terwee, C.B.; Patrick, D.L.; Alonso, J.; Stratford, P.W.; Knol, D.L.; Bouter, L.M.; de Vet, H.C.W. The COSMIN checklist for assessing the methodological quality of studies on measurement properties of health status measurement instruments: An international Delphi study. *Qual. Life Res. Int. J. Qual. Life Asp. Treat. Care Rehabil.* **2010**, *19*, 539–549, doi:10.1007/s11136-010-9606-8.
31. Bot, S.D.M.; Terwee, C.B.; van der Windt, D. a. W.M.; Bouter, L.M.; Dekker, J.; de Vet, H.C.W. Clinimetric evaluation of shoulder disability questionnaires: A systematic review of the literature. *Ann. Rheum. Dis.* **2004**, *63*, 335–341, doi:10.1136/ard.2003.007724.
32. Terwee, C.B.; Bot, S.D.M.; de Boer, M.R.; van der Windt, D.A.W.M.; Knol, D.L.; Dekker, J.; Bouter, L.M.; de Vet, H.C.W. Quality criteria were proposed for measurement properties of health status questionnaires. *J. Clin. Epidemiol.* **2007**, *60*, 34–42, doi:10.1016/j.jclinepi.2006.03.012.
33. Winters, J.D.; Christiansen, C.L.; Stevens-Lapsley, J.E. Preliminary investigation of rate of torque development deficits following total knee arthroplasty. *Knee* **2014**, *21*, 382–386, doi:10.1016/j.knee.2013.10.003.
34. Smith, J.W.; Marcus, R.L.; Peters, C.L.; Pelt, C.E.; Tracy, B.L.; LaStayo, P.C. Muscle force steadiness in older adults before and after total knee arthroplasty. *J. Arthroplast.* **2014**, *29*, 1143–1148, doi:10.1016/j.arth.2013.11.023.
35. Bade, M.J.; Kittelson, J.M.; Kohrt, W.M.; Stevens-Lapsley, J.E. Predicting functional performance and range of motion outcomes after total knee arthroplasty. *Am. J. Phys. Med. Rehabil.* **2014**, *93*, 579–585, doi:10.1097/PHM.0000000000000065.
36. Abdel, M.P.; Parratte, S.; Blanc, G.; Ollivier, M.; Pomero, V.; Viehweger, E.; Argenson, J.-N.A. No benefit of patient–specific instrumentation in TKA on functional and gait outcomes: A randomized clinical trial. *Clin. Orthop.* **2014**, *472*, 2468–2476, doi:10.1007/s11999-014-3544-7.
37. Jenkins, D.; Rodriguez, J.; Ranawat, A.; Alexiades, M.; Deshmukh, A.; Fukunaga, T.; Greiz, M.; Rathod, P.; McHugh, M. A randomized, controlled, prospective study evaluating the effect of patellar eversion on

functional outcomes in primary total knee arthroplasty. *J. Bone Jt. Surg. Am.* **2014**, *96*, 851–858, doi:10.2106/JBJS.L.01513.

38. Thomas, A.C.; Judd, D.L.; Davidson, B.S.; Eckhoff, D.G.; Stevens-Lapsley, J.E. Quadriceps/hamstrings co-activation increases early after total knee arthroplasty. *Knee* **2014**, *21*, 1115–1119, doi:10.1016/j.knee.2014.08.001.
39. Huber, E.O.; Roos, E.M.; Meichtry, A.; de Bie, R.A.; Bischoff-Ferrari, H.A. Effect of preoperative neuromuscular training (NEMEX-TJR) on functional outcome after total knee replacement: An assessor–blinded randomized controlled trial. *BMC Musculoskelet. Disord.* **2015**, *16*, 101, doi:10.1186/s12891-015-0556-8.
40. Calatayud, J.; Casaña, J.; Ezzatvar, Y.; Jakobsen, M.D.; Sundstrup, E.; Andersen, L.L. High–intensity preoperative training improves physical and functional recovery in the early post–operative periods after total knee arthroplasty: A randomized controlled trial. *Knee Surg. Sports Traumatol. Arthrosc. Off. J. ESSKA* **2017**, *25*, 2864–2872, doi:10.1007/s00167-016-3985-5.
41. Bistolfi, A.; Bettoni, E.; Aprato, A.; Milani, P.; Berchialla, P.; Graziano, E.; Massazza, G.; Lee, G.C. The presence and influence of mild depressive symptoms on post–operative pain perception following primary total knee arthroplasty. *Knee Surg. Sports Traumatol. Arthrosc. Off. J. ESSKA* **2017**, *25*, 2792–2800, doi:10.1007/s00167-015-3737-y.
42. Hadlandsmyth, K.; Sabic, E.; Zimmerman, M.B.; Sluka, K.A.; Herr, K.A.; Clark, C.R.; Noiseux, N.O.; Callaghan, J.J.; Geasland, K.M.; Embree, J.L.; et al. Relationships among pain intensity, pain–related distress, and psychological distress in pre–surgical total knee arthroplasty patients: A secondary analysis. *Psychol. Health Med.* **2017**, *22*, 552–563, doi:10.1080/13548506.2016.1189581.
43. Cooper, N.A.; Rakel, B.A.; Zimmerman, B.; Tonelli, S.M.; Herr, K.A.; Clark, C.R.; Noiseux, N.O.; Callaghan, J.J.; Sluka, K.A. Predictors of multidimensional functional outcomes after total knee arthroplasty. *J. Orthop. Res. Off. Publ. Orthop. Res. Soc.* **2017**, *35*, 2790–2798, doi:10.1002/jor.23596.
44. Bonnefoy-Mazure, A.; Martz, P.; Armand, S.; Sagawa, Y.; Suva, D.; Turcot, K.; Miozzari, H.H.; Lübbeke, A. Influence of Body Mass Index on Sagittal Knee Range of Motion and Gait Speed Recovery 1-Year After Total Knee Arthroplasty. *J. Arthroplast.* **2017**, *32*, 2404–2410, doi:10.1016/j.arth.2017.03.008.
45. Loyd, B.J.; Jennings, J.M.; Judd, D.L.; Kim, R.H.; Wolfe, P.; Dennis, D.A.; Stevens-Lapsley, J.E. Influence of Hip Abductor Strength on Functional Outcomes Before and After Total Knee Arthroplasty: Post Hoc Analysis of a Randomized Controlled Trial. *Phys. Ther.* **2017**, *97*, 896–903, doi:10.1093/ptj/pzx066.
46. Lin, Y.-H.; Lee, S.-Y.; Su, W.-R.; Kao, C.-C.; Tai, T.-W.; Chen, T.-B. Effects of nurse-led lower extremity strength training on knee function recovery in patients who underwent total knee replacement. *J. Clin. Nurs.* **2018**, *27*, 1836–1845, doi:10.1111/jocn.14368.
47. Husby, V.S.; Foss, O.A.; Husby, O.S.; Winther, S.B. Randomized controlled trial of maximal strength training vs. standard rehabilitation following total knee arthroplasty. *Eur. J. Phys. Rehabil. Med.* **2018**, *54*, 371–379, doi:10.23736/S1973-9087.17.04712-8.
48. Paravlic, A.H.; Pisot, R.; Marusic, U. Specific and general adaptations following motor imagery practice focused on muscle strength in total knee arthroplasty rehabilitation: A randomized controlled trial. *PLoS ONE* **2019**, *14*, e0221089, doi:10.1371/journal.pone.0221089.
49. Indelli, P.F.; Risitano, S.; Hall, K.E.; Leonardi, E.; Migliore, E. Effect of polyethylene conformity on total knee arthroplasty early clinical outcomes. *Knee Surg. Sports Traumatol. Arthrosc. Off. J. ESSKA* **2019**, *27*, 1028–1034, doi:10.1007/s00167-018-5170-5.
50. Jiang, H.-H.; Jian, X.-F.; Shangguan, Y.-F.; Qing, J.; Chen, L.-B. Effects of Enhanced Recovery After Surgery in Total Knee Arthroplasty for Patients Older Than 65 Years. *Orthop. Surg.* **2019**, *11*, 229–235, doi:10.1111/os.12441.
51. Liljensøe, A.; Laursen, J.O.; Bliddal, H.; Søballe, K.; Mechlenburg, I. Weight Loss Intervention Before Total Knee Replacement: A 12-Month Randomized Controlled Trial. *Scand. J. Surg. SJS Off. Organ. Finn. Surg. Soc. Scand. Surg. Soc.* **2019**, 1457496919883812, doi:10.1177/1457496919883812.
52. Skoffer, B.; Maribo, T.; Mechlenburg, I.; Korsgaard, C.G.; Søballe, K.; Dalgas, U. Efficacy of preoperative progressive resistance training in patients undergoing total knee arthroplasty: 12–month follow–up data from a randomized controlled trial. *Clin. Rehabil.* **2019**, *34*, 82–90, doi:10.1177/0269215519883420.
53. Roos, E.M.; Toksvig-Larsen, S. Knee injury and Osteoarthritis Outcome Score (KOOS)—validation and comparison to the WOMAC in total knee replacement. *Health Qual. Life Outcomes* **2003**, *1*, 17, doi:10.1186/1477-7525-1-17.

54. de Groot, I.B.; Favejee, M.M.; Reijman, M.; Verhaar, J.A.; Terwee, C.B. The Dutch version of the knee injury and osteoarthritis outcome score: A validation study. *Health Qual. Life Outcomes* **2008**, *6*, 16, doi:10.1186/1477-7525-6-16.
55. Gandek, B.; Ware, J.E. Validity and Responsiveness of the Knee Injury and Osteoarthritis Outcome Score: A Comparative Study Among Total Knee Replacement Patients. *Arthritis Care Res.* **2017**, *69*, 817–825, doi:10.1002/acr.23193.
56. Roos, E.M.; Lohmander, L.S. The Knee injury and Osteoarthritis Outcome Score (KOOS): From joint injury to osteoarthritis. *Health Qual. Life Outcomes* **2003**, *1*, 64, doi:10.1186/1477-7525-1-64.
57. Ornetti, P.; Parratte, S.; Gossec, L.; Tavernier, C.; Argenson, J.-N.; Roos, E.M.; Guillemin, F.; Maillefert, J.F. Cross-cultural adaptation and validation of the French version of the Knee injury and Osteoarthritis Outcome Score (KOOS) in knee osteoarthritis patients. *Osteoarthr. Cartil.* **2008**, *16*, 423–428, doi:10.1016/j.joca.2007.08.007.
58. Bombardier, C.; Melfi, C.A.; Paul, J.; Green, R.; Hawker, G.; Wright, J.; Coyte, P. Comparison of a generic and a disease–specific measure of pain and physical function after knee replacement surgery. *Med. Care* **1995**, *33*, AS131–CAS144.
59. McConnell, S.; Kolopack, P.; Davis, A.M. The Western Ontario and McMaster Universities Osteoarthritis Index (WOMAC): A review of its utility and measurement properties. *Arthritis Rheum.* **2001**, *45*, 453–461, doi:10.1002/1529-0131(200110)45:5<453::aid-art365>3.0.co;2-w.
60. Angst, F.; Aeschlimann, A.; Stucki, G. Smallest detectable and minimal clinically important differences of rehabilitation intervention with their implications for required sample sizes using WOMAC and SF–36 quality of life measurement instruments in patients with osteoarthritis of the lower extremities. *Arthritis Rheum.* **2001**, *45*, 384–391, doi:10.1002/1529-0131(200108)45:4<384::AID-ART352>3.0.CO;2-0.
61. Dunbar, M.J.; Robertsson, O.; Ryd, L.; Lidgren, L. Appropriate questionnaires for knee arthroplasty. Results of a survey of 3600 patients from The Swedish Knee Arthroplasty Registry. *J. Bone Jt. Surg. Br.* **2001**, *83*, 339–344, doi:10.1302/0301-620x.83b3.11134.
62. Stratford, P.W.; Kennedy, D.M.; Woodhouse, L.J.; Spadoni, G.F. Measurement properties of the WOMAC LK 3.1 pain scale. *Osteoarthr. Cartil.* **2007**, *15*, 266–272, doi:10.1016/j.joca.2006.09.005.
63. Noble, P.C.; Scuderi, G.R.; Brekke, A.C.; Sikorskii, A.; Benjamin, J.B.; Lonner, J.H.; Chadha, P.; Daylamani, D.A.; Scott, W.N.; Bourne, R.B. Development of a new Knee Society scoring system. *Clin. Orthop.* **2012**, *470*, 20–32, doi:10.1007/s11999-011-2152-z.
64. Culliton, S.E.; Bryant, D.M.; MacDonald, S.J.; Hibbert, K.M.; Chesworth, B.M. Validity and Internal Consistency of the New Knee Society Knee Scoring System. *Clin. Orthop.* **2018**, *476*, 77–84, doi:10.1007/s11999.0000000000000014.
65. Dawson, J.; Fitzpatrick, R.; Murray, D.; Carr, A. Questionnaire on the perceptions of patients about total knee replacement. *J. Bone Jt. Surg. Br.* **1998**, *80-B*, 63–69, doi:10.1302/0301-620X.80B1.0800063.
66. Harris, K.; Dawson, J.; Gibbons, E.; Lim, C.R.; Beard, D.J.; Fitzpatrick, R.; Price, A.J. Systematic review of measurement properties of patient-reported outcome measures used in patients undergoing hip and knee arthroplasty. *Patient Relat. Outcome Meas.* **2016**, *7*, 101–108, doi:10.2147/PROM.S97774.
67. Xie, F.; Ye, H.; Zhang, Y.; Liu, X.; Lei, T.; Li, S.-C. Extension from inpatients to outpatients: Validity and reliability of the Oxford Knee Score in measuring health outcomes in patients with knee osteoarthritis. *Int. J. Rheum. Dis.* **2011**, *14*, 206–210, doi:10.1111/j.1756-185X.2010.01580.x.
68. Beard, D.J.; Harris, K.; Dawson, J.; Doll, H.; Murray, D.W.; Carr, A.J.; Price, A.J. Meaningful changes for the Oxford hip and knee scores after joint replacement surgery. *J. Clin. Epidemiol.* **2015**, *68*, 73–79, doi:10.1016/j.jclinepi.2014.08.009.
69. Jourdan, C.; Poiraudeau, S.; Descamps, S.; Nizard, R.; Hamadouche, M.; Anract, P.; Boisgard, S.; Galvin, M.; Ravaud, P. Comparison of patient and surgeon expectations of total hip arthroplasty. *PLoS ONE* **2012**, *7*, e30195, doi:10.1371/journal.pone.0030195.
70. Neuprez, A.; Delcour, J.-P.; Fatemi, F.; Gillet, P.; Mawet, M.; François, G.; Bruyère, O.; Crielaard, J.-M.; Gosset, C.; Reginster, J.-Y. Development and validation of the French version of a tool assessing patient's expectations in lower limb osteoarthritis. *J. Orthop.* **2015**, *12*, 46–57, doi:10.1016/j.jor.2014.06.002.
71. Julian, L.J. Measures of anxiety: State-Trait Anxiety Inventory (STAI), Beck Anxiety Inventory (BAI), and Hospital Anxiety and Depression Scale-Anxiety (HADS–A). *Arthritis Care Res.* **2011**, *63*, S467-472, doi:10.1002/acr.20561.

72. Smarr, K.L.; Keefer, A.L. Measures of depression and depressive symptoms: Beck Depression Inventory–II (BDI–II), Center for Epidemiologic Studies Depression Scale (CES–D), Geriatric Depression Scale (GDS), Hospital Anxiety and Depression Scale (HADS), and Patient Health Questionnaire–9 (PHQ–9). *Arthritis Care Res.* **2011**, *63*, S454–466, doi:10.1002/acr.20556.
73. Axford, J.; Butt, A.; Heron, C.; Hammond, J.; Morgan, J.; Alavi, A.; Bolton, J.; Bland, M. Prevalence of anxiety and depression in osteoarthritis: Use of the Hospital Anxiety and Depression Scale as a screening tool. *Clin. Rheumatol.* **2010**, *29*, 1277–1283, doi:10.1007/s10067-010-1547-7.
74. Bjelland, I.; Dahl, A.A.; Haug, T.T.; Neckelmann, D. The validity of the Hospital Anxiety and Depression Scale. An updated literature review. *J. Psychosom. Res.* **2002**, *52*, 69–77, doi:10.1016/s0022-3999(01)00296-3.
75. Spinhoven, P.; Ormel, J.; Sloekers, P.P.; Kempen, G.I.; Speckens, A.E.; Van Hemert, A.M. A validation study of the Hospital Anxiety and Depression Scale (HADS) in different groups of Dutch subjects. *Psychol. Med.* **1997**, *27*, 363–370, doi:10.1017/s0033291796004382.
76. Perneger, T.V.; Leplège, A.; Etter, J.F.; Rougemont, A. Validation of a French–language version of the MOS 36–Item Short Form Health Survey (SF-36) in young healthy adults. *J. Clin. Epidemiol.* **1995**, *48*, 1051–1060, doi:10.1016/0895-4356(94)00227-h.
77. Webster, K.E.; Feller, J.A. Comparison of the short form–12 (SF–12) health status questionnaire with the SF–36 in patients with knee osteoarthritis who have replacement surgery. *Knee Surg. Sports Traumatol. Arthrosc. Off. J. ESSKA* **2016**, *24*, 2620–2626, doi:10.1007/s00167-015-3904-1.
78. Escobar, A.; Quintana, J.M.; Bilbao, A.; Aróstegui, I.; Lafuente, I.; Vidaurreta, I. Responsiveness and clinically important differences for the WOMAC and SF-36 after total knee replacement. *Osteoarthr. Cartil.* **2007**, *15*, 273–280, doi:10.1016/j.joca.2006.09.001.
79. Conner-Spady, B.L.; Marshall, D.A.; Bohm, E.; Dunbar, M.J.; Noseworthy, T.W. Comparing the validity and responsiveness of the EQ–5D–5L to the Oxford hip and knee scores and SF-12 in osteoarthritis patients 1 year following total joint replacement. *Qual. Life Res. Int. J. Qual. Life Asp. Treat. Care Rehabil.* **2018**, *27*, 1311–1322, doi:10.1007/s11136-018-1808-5.
80. Gandhi, S.K.; Salmon, J.W.; Zhao, S.Z.; Lambert, B.L.; Gore, P.R.; Conrad, K. Psychometric evaluation of the 12–item short–form health survey (SF–12) in osteoarthritis and rheumatoid arthritis clinical trials. *Clin. Ther.* **2001**, *23*, 1080–1098, doi:10.1016/s0149-2918(01)80093-x.
81. Youngcharoen, P.; Aree-Ue, S.; Saraboon, Y. Validation of pain catastrophizing scale Thai version in older adults with knee osteoarthritis. *Innov. Aging* **2017**, *1*, 871–871, doi:10.1093/geroni/igx004.3131.
82. Alghadir, A.H.; Anwer, S.; Iqbal, A.; Iqbal, Z.A. Test–retest reliability, validity, and minimum detectable change of visual analog, numerical rating, and verbal rating scales for measurement of osteoarthritic knee pain. *J. Pain Res.* **2018**, *11*, 851–856, doi:10.2147/JPR.S158847.
83. Rogers, J.C.; Irrgang, J.J. Measures of adult lower extremity function: The American Academy of Orthopedic Surgeons Lower Limb Questionnaire, The Activities of Daily Living Scale of the Knee Outcome Survey (ADLS), Foot Function Index (FFI), Functional Assessment System (FAS), Harris Hip Score (HHS), Index of Severity for Hip Osteoarthritis (ISH), Index of Severity for Knee Osteoarthritis (ISK), Knee Injury and Osteoarthritis Outcome Score (KOOS), and Western Ontario and McMaster Universities Osteoarthritis Index (WOMAC™). *Arthritis Care Res.* **2003**, *49*, S67–S84, doi:10.1002/art.11401.
84. Unver, B.; Kalkan, S.; Yuksel, E.; Kahraman, T.; Karatosun, V. Reliability of the 50–foot walk test and 30–sec chair stand test in total knee arthroplasty. *Acta Ortop. Bras.* **2015**, *23*, 184–187, doi:10.1590/1413-78522015230401018.
85. Bellamy, N. Outcome measurement in osteoarthritis clinical trials. *J. Rheumatol. Suppl.* **1995**, *43*, 49–51.
86. Mutlu, E.K.; Ozdincler, A.R. Reliability and responsiveness of algometry for measuring pressure pain threshold in patients with knee osteoarthritis. *J. Phys. Ther. Sci.* **2015**, *27*, 1961–1965, doi:10.1589/jpts.27.1961.
87. Lienhard, K.; Lauermann, S.P.; Schneider, D.; Item-Glatthorn, J.F.; Casartelli, N.C.; Maffiuletti, N.A. Validity and reliability of isometric, isokinetic and isoinertial modalities for the assessment of quadriceps muscle strength in patients with total knee arthroplasty. *J. Electromyogr. Kinesiol. Off. J. Int. Soc. Electrophysiol. Kinesiol.* **2013**, *23*, 1283–1288, doi:10.1016/j.jelekin.2013.09.004.
88. Tolk, J.J.; Janssen, R.P.A.; Prinsen, C. a. C.; Latijnhouwers, D.A.J.M.; van der Steen, M.C.; Bierma-Zeinstra, S.M.A.; Reijman, M. The OARSI core set of performance-based measures for knee osteoarthritis is reliable but not valid and responsive. *Knee Surg. Sports Traumatol. Arthrosc. Off. J. ESSKA* **2019**, *27*, 2898–2909, doi:10.1007/s00167-017-4789-y.

89. Ateef, M.; Kulandaivelan, S.; Tahseen, S. Test–Retest Reliability and Correlates of 6–Minute Walk Test in Patients with Primary Osteoarthritis of Knees. Available online: https://link.galegroup.com/apps/doc/A578163738/AONE?sid=lms (accessed on 2 January 2020).
90. Lavernia, C.; D'Apuzzo, M.; Rossi, M.D.; Lee, D. Accuracy of knee range of motion assessment after total knee arthroplasty. *J. Arthroplast.* **2008**, *23*, 85–91, doi:10.1016/j.arth.2008.05.019.
91. Kennedy, D.M.; Stratford, P.W.; Wessel, J.; Gollish, J.D.; Penney, D. Assessing stability and change of four performance measures: A longitudinal study evaluating outcome following total hip and knee arthroplasty. *BMC Musculoskelet. Disord.* **2005**, *6*, 3, doi:10.1186/1471-2474-6-3.
92. Yuksel, E.; Kalkan, S.; Cekmece, S.; Unver, B.; Karatosun, V. Assessing Minimal Detectable Changes and Test–Retest Reliability of the Timed Up and Go Test and the 2–Minute Walk Test in Patients With Total Knee Arthroplasty. *J. Arthroplast.* **2017**, *32*, 426–430, doi:10.1016/j.arth.2016.07.031.
93. Ishii, Y.; Noguchi, H.; Sato, J.; Ishii, H.; Yamamoto, T.; Sakurai, T.; Toyabe, S.-I. Clinical relevance of active straight leg raising, standing up, and walking after total knee arthroplasty in a cross–sectional study. *Eur. J. Orthop. Surg. Traumatol. Orthop. Traumatol.* **2018**, *28*, 947–953, doi:10.1007/s00590-017-2100-z.
94. Alghadir, A.; Anwer, S.; Brismée, J.-M. The reliability and minimal detectable change of Timed Up and Go test in individuals with grade 1–3 knee osteoarthritis. *BMC Musculoskelet. Disord.* **2015**, *16*, 174, doi:10.1186/s12891-015-0637-8.
95. Dobson, F.; Hinman, R.S.; Roos, E.M.; Abbott, J.H.; Stratford, P.; Davis, A.M.; Buchbinder, R.; Snyder-Mackler, L.; Henrotin, Y.; Thumboo, J.; et al. OARSI recommended performance-based tests to assess physical function in people diagnosed with hip or knee osteoarthritis. *Osteoarthr. Cartil.* **2013**, *21*, 1042–1052, doi:10.1016/j.joca.2013.05.002.
96. Bagby, R.M.; Ryder, A.G.; Schuller, D.R.; Marshall, M.B. The Hamilton Depression Rating Scale: Has the gold standard become a lead weight? *Am. J. Psychiatry* **2004**, *161*, 2163–2177, doi:10.1176/appi.ajp.161.12.2163.
97. Steffen, T.M.; Hacker, T.A.; Mollinger, L. Age–and gender–related test performance in community–dwelling elderly people: Six–Minute Walk Test, Berg Balance Scale, Timed Up & Go Test, and gait speeds. *Phys. Ther.* **2002**, *82*, 128–137.
98. Huber, E.O.; Meichtry, A.; de Bie, R.A.; Bastiaenen, C.H. Construct validity of change scores of the Chair Stand Test versus Timed Up and Go Test, KOOS questionnaire and the isometric muscle strength test in patients with severe knee osteoarthritis undergoing total knee replacement. *Man. Ther.* **2016**, *21*, 262–267, doi:10.1016/j.math.2015.09.012.
99. Medina-Mirapeix, F.; Vivo-Fernández, I.; López-Cañizares, J.; García-Vidal, J.A.; Benítez-Martínez, J.C.; Del Baño-Aledo, M.E. Five times sit-to-stand test in subjects with total knee replacement: Reliability and relationship with functional mobility tests. *Gait Posture* **2018**, *59*, 258–260, doi:10.1016/j.gaitpost.2017.10.028.
100. Roussel, N.A.; Nijs, J.; Truijen, S.; Smeuninx, L.; Stassijns, G. Low back pain: Clinimetric properties of the Trendelenburg test, active straight leg raise test, and breathing pattern during active straight leg raising. *J. Manip. Physiol. Ther.* **2007**, *30*, 270–278, doi:10.1016/j.jmpt.2007.03.001.
101. de Vet, H.C.W.; Terwee, C.B.; Knol, D.L.; Bouter, L.M. When to use agreement versus reliability measures. *J. Clin. Epidemiol.* **2006**, *59*, 1033–1039, doi:10.1016/j.jclinepi.2005.10.015.
102. Jakobsen, T.L.; Christensen, M.; Christensen, S.S.; Olsen, M.; Bandholm, T. Reliability of knee joint range of motion and circumference measurements after total knee arthroplasty: Does tester experience matter? *Physiother. Res. Int. J. Res. Clin. Phys. Ther.* **2010**, *15*, 126–134, doi:10.1002/pri.450.
103. Motyl, J.M.; Driban, J.B.; McAdams, E.; Price, L.L.; McAlindon, T.E. Test–retest reliability and sensitivity of the 20–meter walk test among patients with knee osteoarthritis. *BMC Musculoskelet. Disord.* **2013**, *14*, 166, doi:10.1186/1471-2474-14-166.
104. Ornetti, P.; Maillefert, J.-F.; Laroche, D.; Morisset, C.; Dougados, M.; Gossec, L. Gait analysis as a quantifiable outcome measure in hip or knee osteoarthritis: A systematic review. *Jt. Bone Spine Rev. Rhum.* **2010**, *77*, 421–425, doi:10.1016/j.jbspin.2009.12.009.
105. Almeida, G.J.; Irrgang, J.J.; Fitzgerald, G.K.; Jakicic, J.M.; Piva, S.R. Reliability of Physical Activity Measures During Free–Living Activities in People After Total Knee Arthroplasty. *Phys. Ther.* **2016**, *96*, 898–907, doi:10.2522/ptj.20150407.

© 2020 by the authors. Licensee MDPI, Basel, Switzerland. This article is an open access article distributed under the terms and conditions of the Creative Commons Attribution (CC BY) license (http://creativecommons.org/licenses/by/4.0/).
